# Clinical and molecular features of an infant patient affected by Leigh Disease associated to m.14459G > A mitochondrial DNA mutation: a case report

**DOI:** 10.1186/1471-2377-11-85

**Published:** 2011-07-12

**Authors:** Dario Ronchi, Alessandra Cosi, Davide Tonduti, Simona Orcesi, Andreina Bordoni, Francesco Fortunato, Mafalda Rizzuti, Monica Sciacco, Martina Collotta, Sophie Cagdas, Giuseppe Capovilla, Maurizio Moggio, Angela Berardinelli, Pierangelo Veggiotti, Giacomo P Comi

**Affiliations:** 1 Centro Dino Ferrari, Department of Neurological Sciences, University of Milan, Foundation IRCCS Ca' Granda Ospedale Maggiore Policlinico, via Francesco Sforza 35, 20122 Milan, Italy; 2 Foundation IRCCS Istituto Neurologico C. Mondino, Via Mondino 2, 27100 Pavia, Italy; 3 Epilepsy Center, Department of Child Neuropsychiatry, C Poma Hospital, Strada Lago Paiolo 10, 46100 Mantova, Italy

**Keywords:** Leigh Syndrome, mitochondrial DNA, LHON, MT-ND6, MT-CYB, mitochondrial Complex I

## Abstract

**Background:**

Leigh Syndrome (LS) is a severe neurodegenerative disorder characterized by bilateral symmetrical necrotic lesions in the basal ganglia and brainstem. Onset is in early infancy and prognosis is poor. Causative mutations have been disclosed in mitochondrial DNA and nuclear genes affecting respiratory chain subunits and assembly factors.

**Case presentation:**

Here we report the clinical and molecular features of a 15-month-old female LS patient. Direct sequencing of her muscle-derived mtDNA revealed the presence of two apparently homoplasmic variants: the novel m.14792C > G and the already known m.14459G > A resulting in p.His16Asp change in cytochrome b (MT-CYB) and p.Ala72Val substitution in ND6 subunit, respectively. The m.14459G > A was heteroplasmic in the mother's blood-derived DNA.

**Conclusions:**

The m.14459G > A might lead to LS, complicated LS or Leber Optic Hereditary Neuropathy. A comprehensive re-evaluation of previously described 14459G > A-mutated patients does not explain this large clinical heterogeneity.

## 1. Background

Leigh Syndrome (LS, OMIM 256000) is a subacute necrotizing encephalomyelopathy characterized by bilateral symmetrical necrotic lesions of gray matter nuclei in the basal ganglia, diencephalon, cerebellum or brainstem. The onset is usually in early infancy and patients manifest an heterogeneous set of symptoms, such as regression or psychomotor delay, weakness, hypotonia, truncal ataxia, intention tremor associated with lactic acidosis in the blood, cerebrospinal fluid or urine. The prognosis is poor and, in most cases, patients die before age of 5 years. It is the most frequent cause of inherited mitochondrial disorder in infancy (1:40,000) [1].

LS inheritance is complex since patients may present mutations in mitochondrial DNA (mtDNA) or in nuclear genes, which predominantly encode for proteins involved in respiratory chain structure and assembly or in coenzyme Q10 biogenesis [2]. Among maternally inherited forms, most of the mutations lay within genes encoding Complex I (ND1-6) and V (ATP6, ATP8) mitochondrial subunits [3].

Here we report the clinical, biochemical and molecular features of a pediatric patient affected by Leigh Syndrome associated with the mitochondrial DNA mutation m.14459G > A within MT-ND6 gene.

## 2. Case Presentation

### Case report

**Figure 1:**
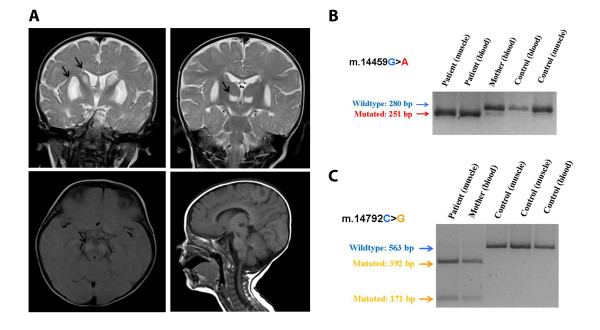
**Neuroradiological and molecular features in our proband**. **A**. Brain MRI showed bilateral symmetric hyperintense lesions on T2-weighted images in the head of caudate, putamen (top left), thalami (top right) and ventral mesencephalum (bottom left); these lesions were hypointense in T1-weighted images (bottom right). **B**. PCR-RFLP analysis of m.14459G > A mutation. The transition m.14459G > A creates a restriction site for endonuclease MaeIII in mutated amplicons obtained using a modified primer set previously described (FOR14430*-RC14710) producing two fragments of 251 and 29 base pairs (the latter is not visible on the agarose gel) while wild type molecules remain uncut. **C**. PCR-RFLP analysis of m.14792C > G variant. The variant m.14792C > G is recognized by restriction endonuclease HinfI which cuts mutated PCR-amplified fragments (encompassing 14400-14963 nucleotides) producing molecules of 392 and 171 base pairs. Control samples are from healthy unrelated independent subjects.

The proband is a 15-month-old female patient born from healthy nonconsanguineous Italian parents after a regular pregnancy and delivery. Feeding problems and gastroesophageal reflux were noted from the first weeks of life. At the age of 3 months, after the first vaccination, she started to present dystonic postures of the trunk initially misdiagnosed as related to gastroesophageal reflux. At the age of 5 months, after the second vaccination, she started to present irritability, inconsolable crying, psychomotor regression and generalized seizures. She was treated with endorectal diazepam, and after that she had no others seizure episodes. At 7 months of age, after an intercurrent viral illness, she was referred to our department in Pavia. The clinical picture was characterized by psychomotor delay, dystonic-dyskinetic tetraparesis, startle reaction to sudden acoustic stimuli, episodic central apneas. Plasma amino acids revealed high level of alanine, valine, isoleucine and lysine with abnormal ratio alanine:lysine (3.7, range of normality = 1.5-2.5). Urinary organic acids revealed high level of lactic, pyruvic, fumaric and 3-ketoglutaric acids. High levels of lactic and pyruvic acids were also demonstrated both in plasma and CSF. MRI showed bilateral symmetric hyperintense lesions on T2-weighted images in the basal ganglia, thalami and ventral mesencephalum (Figure 1A). Brain CT scan didn't show calcification. Fundus oculi showed retinal depigmentation; assessment of VEPs revealed evidence of a conduction delay and ERG showed retinal involvement. Auditory brainstem responses, ECG and echocardiogram were normal.

A treatment with riboflavine 50 mg/day, thiamine 150 mg/day 300 mg: 1/2 cp, carnitine 100 mg/kg/day was started.

At 8 months of age she started to present external ophthalmoplegia and absence of pupillary reaction. Hereafter the clinical picture remained stable.

A muscle tissue biopsy was normal at the morphological and histochemical studies, with the exception of a mild increase in acid phosphatase staining at subsarcolemmal level in a few fibres.

Family history for neurological disorders was negative. General clinical assessment of her mother was normal.

### Results

Spectrophotometric determination of the activity of mitochondrial Complex I in muscle showed a residual activity of 36% compared to controls (CI/CS = 5.9 nM/min/mg protein, control values = 16.5 ± 2.6 normalized to citrate synthase, CS).

Large-scale mtDNA rearrangements and mtDNA depletion was excluded by Southern Blot and quantitative PCR assays on muscle-derived DNA.

Direct sequencing of muscle-derived mtDNA revealed the presence of two apparently homoplasmic variants: the novel m.14792C > G and the already known m.14459G > A resulting in p.His16Asp change in cytochrome b (MT-CYB) and p.Ala72Val substitution in ND6 subunit, respectively. Their presence was confirmed by specific PCR-RFLP assays. The sequence also showed that the patient belongs to the mitochondrial haplogroup H2a2, consisting to European ethnic groups.

The mother's mtDNA was homoplasmic for MT-CYB variant and heteroplasmic for the MT-ND6 mutation with a mutational load estimated less than 2% by PCR-RFLP analysis (Figure 1B and Figure 1C).

## 3. Conclusions

The variant m.14459G > A has been so far reported in six patients affected by Leber's hereditary optic neuropathy (LHON), in seven probands mainly showing dystonia and in four LS/Leigh-like Syndrome (LLS) patients [4-9]. Clinical, biochemical and genetical features of the aforementioned probands are summarized in Table 1. Phenotypic manifestations of this variant are expressed in both sexes with approximately equal frequency. However, the reason for the striking difference in the clinical outcome of the m.14459G > A mutation between patients is unknown.

**Table 1:** Clinical, biochemical and molecular features of m.14459G > A mutated patients:

Reference	Patient	Sex	Ethnic group	Age	Age of Onset	Phenotype	Clinical features	Neuroradiology	Residual CI activity	m.14459G > A Mutational load (%)
Current Study	A	F	Caucasian Haplogroup H2a2	18 m	2 m	LS+Dystonia	Psycomotor delay, hypotonia, dystonia.	MRI: lesions in the basal ganglia and mesencephalum.	36% (muscle)	> 99 (muscle) >99 (blood)

Shoffner et al. [5]	B1-1 (proband)	F	African-American	19y	3 m	LHON	Loss of bilateral vision, optic atrophy	MRI: lesions in caudate nucleus and right putamen	3% (muscle)	>99

Shoffner et al. [5]	B1-2 (proband's mother)	F	African-American	42y	42y	LHON	Loss of bilateral vision, optic atrophy	NP	NP	50

Shoffner et al. [5]	B2	F	Caucasian	13y	3y	Dystonia	Dysarthria, dystonia, quadriparesis	CT: bilateral hipodensity in the putamina. MRI: bilateral lesions in putamen and caudate.	NP	50

Funalot et al. [7]	C2	M	Caucasian Haplogroup T	†32y suicide	18y	LLS+LHON	Bilateral visual failure, hearing loss, ataxia, extrapiramidal signs	MRI: hyperintensity in dorsal midbrain; bilateral lesions in the third ventricle walls.	NP	40

Gropman et al. [9]	D1 (proband)	F	Hispanic	4y	3y	Dystonia	Anarthria, dystonia, spasticity, encefalopathy, neurofibromatosis	MRI: bilateral hyperintensities of the putamen. MRS: elevated lactate.	76% (muscle)	>99

Gropman et al. [9]	D1-2 (proband's mother)	F	Hispanic	35y	34y		neurofibromatosis, muscle weakness	MRI: bitemporal signal abnormalities	NP	>99

Gropman et al. [9]	D1-3 (proband's brother)	M	Hispanic	7y	2y	Dystonia	neurofibromatosis, hemiparesis, cognitive delay	MRI: right sided putaminal infarcts, thickened corpus callosum	NP	>99

Gropman et al. [9]	D1-3 (maternal cousin)	F	Hispanic	5y	5y	-	Hemiparesis	MRI: bilateral putaminal infarcts MRS: elevated lactate	NP	>99

Kirby et al. [6]	E1-1	F	?	†7y	9 m	LS	Motor delay, hypotonia, seizure, dystonia.	CT: bilateral low density lesions of the basal ganglia	25% (fibroblasts)	97 (fibroblasts)

Kirby et al. [6]	E1-2 (proband's brother)	M	?	†10 m	3 m	LS	Hypotonia, neurological and developmental regression, seizure. Metabolic acidosis.	-	6% (muscle) 46% (liver)	97 (muscle, liver)

Kirby et al. [6]	E2	F	?	†8 m	6 m	LS	Developmental delay, athetosis, hypotonia.	MRI: cerebral atrophy, involvement of basal ganglia and medial thalamic nuclei.	15% (fibroblasts) 16% (muscle) 37% (liver)	97 (muscle, liver)

Jun et al. [4]	G1-1 (proband)	F	Hispanic (Native American). Haplogroup D	10y	2y	Dystonia	Dystonia, corticospinal tract dysfunction, dysarthria.	CT: bilateral low density lesions in putamen progressing to the caudate	-	99 (blood)

Jun et al. [4]	G1-2 (proband's brother)	M	Hispanic (Native American). Haplogroup D	13y	13y	Dystonia	Dystonia, intellectual impairmeint.	NP	NP	99 (blood)

Jun et al. [4]	G1-3 (proband's mother)	F	Hispanic (Native American). Haplogroup D	32y	32y	LHON	Bilateral optic atrophy.	NP	NP	73 (blood)

Jun et al. [4]	G1-4 (maternal cousin)	M	Hispanic (Native American). Haplogroup D	?	?	LHON	-	NP	NP	99 (blood)

Jun et al. [4]	G1-5 (maternal relative)	M	Hispanic (Native American). Haplogroup D	?	5y	Dystonia	Dystonia	CT: abnormal basal ganglial lucencies	NP	99 (blood)

Tarnopolsky et al. [8]	E1-1 (proband)	F	Caucasian	45y	8y	Dystonia	Dystonia	MRI: hiperintensities signal in putamen	N	34 (blood)

Tarnopolsky et al. [8]	E1-2 (proband's brother)	M	Caucasian	49y	19y	LHON	Visual loss, hearing loss	NP	NP	18 (blood)

Tarnopolsky et al. [8]	E1-3 (proband's brother)	M	Caucasian	56y	?	-	Visual changes, hearing loss, muscle weakness.	NP	NP	21 (blood)

Tarnopolsky et al. [8]	E1-4 (proband's mother)	F	Caucasian	78y	76y	-	Stroke episodes.	CT: hipodensity in right frontal periventricular region	NP	4 (blood)

Tarnopolsky et al. [8]	E2	M	Caucasian	17y	16y	LHON	Optic nerve pallor, visual loss.	MRI: normal	NP	26 (blood)

NP: not performed; CI: Complex I,% values refer to activity compared to controls.

Patients so far described belong to different ethnic groups and they likely present different mtDNA haplogroups although a precise definition of the haplotypes is available in few subjects. The available haplotypes include H, T (West Eurasia and Middle East) and D (East Asia and Americas). This fact suggests that the mutation arose independently several times in a period of time relatively recent.

Although mitochondrial haplogroups could influence the phenotypic expression of LHON and LS associated variant [10-12], no evidence has been produced for m.14459G > A and haplotype H2a2 disclosed in our patient.

Clinical manifestations of m.14459G > A transition seem to mainly affect central nervous system and skeletal muscle. CNS, brainstem and optic nerve are often compromised but some patients may present more systemic manifestations. Dystonia is the most occurring feature described alone or associated to LS/LLS.

The transition m.14459G > A is considered a rare causative mutation for LHON [13]. Our proband had delayed VEP, together with signs of retinal involvement, probably reflecting multiple sites of affection along the optic pathways. The involvement of visual pathway in 14459G > A-mutated patients is generally a later event in contrast to dystonia that develops in the early childhood. To date the co-existence of LLS and LHON has been clearly described in a single proband [7].

Laboratory findings are inconsistent and the elevation of lactate level in blood and/or CSF found in some LS patients, as wells as in our proband, is not peculiar to this molecular defect.

Neuroradiological examinations, when performed, showed lesions in brainstem and basal ganglia in LS and dystonia patients. A positive result at MRI investigation could address the diagnosis of dystonia or LS although exceptions to this correlation have been reported.

Muscle biopsy did not show striking signs of mitochondrial impairment: in our proband no ragged-red or cytochrome *c *oxidase-negative fibers were observed. Minor changes such as mild variations in fiber size and accumulation of subsarcolemmal mitochondria have been reported in a few patients [5,7,8]. As in the case of other mutations affecting structure or assembly of Complex I, histological analysis may not prove helpful in uncovering mitochondrial pathology.

Spectrophotometric analysis of the activity of respiratory chain complexes has been reported in early onset patients who underwent muscle biopsy. Residual activity of Complex I ranges from 3 to 76% compared to control. Secondary defects have been also observed in Complex III [5]. Fibroblasts culture established from two LS patients confirmed a severe reduction of enzymatic activity [6]. In our patient we observed a strong reduction of Complex I activity in skeletal muscle, compatible with the marked hypotonia observed.

Analysis of transmitochondrial cybrids previously confirmed that m.14459G > A leads to the impairment of Complex I activity [14].

No obvious correlation between mutant load and clinical features is observed. High levels of heteroplasmy in blood have been described in LHON, dystonia and LS patients. In the first report [4], even an asymptomatic subject had 99% of mutant mtDNA in lymphoblasts but it was suggested that it could be due to an artifact of transformation and cell culture [6]. Tissues derived from LS patients generally present a virtually homoplasmic state [6], as in the case of our proband. Conversely, mutant DNA detected in blood from LHON patients differs widely, with heteroplasmy ranging from 18 to 99%.

The pathological manifestation of the m.14459G > A mutation is thought to be highly tissue-specific, depending also on the levels of heteroplasmy present in each organ or tissue involved and this fact could explain the different clinical phenotypes [5]. The existence of secondary mtDNA variants able to modify the disease presentation of a primary mutation has been suggested before [15,16]. In our proband, we observed a novel homoplasmic variant m.14792C > G, which was absent in 162 Italian healthy controls. The proband's mother was homoplasmic for this nucleotide change (and heteroplasmic for the m.14459G > A mutation). Since her general clinical assessment was normal, the MT-CYB variant is unlikely to have a pathogenic significance.

Unfortunately, no cytochrome b region sequence data are available for the previously reported 14459G > A-mutated subjects. Moreover, some of the reported probands, who share the same maternal lineage, present very heterogeneous symptoms [8,9]. These elements suggest that other factors, external to mitochondrial DNA, are relevant to explain the clinical heterogeneity of m.14459 mutation, making the contribution to disease of the novel m.14792C > G variant purely speculative. The assessment of respiratory chain activity in transmitochondrial cybrids derived from the proband and her mother would be helpful to settle this point but this analysis is unfortunately not feasible, due to the unavailability of primary cell cultures.

## Consent

Written informed consent was obtained from the patient's parents for publication of this case report and any accompanying images. A copy of the written consent is available for review by the Editor in Chief of this journal.

## List of abbreviations

LS: Leigh Syndrome; mtDNA: mitochondrial DNA; PCR-RFLP: polymerase chain reaction - restriction site length polymorphism; LHON: Leber's hereditary optic neuropathy; CNS: central nervous system; CSF: cerebrospinal fluid.

## Competing interests

The authors declare that they have no competing interests.

## Authors' contributions

DR and AC participated the molecular genetic studies and drafted the manuscript. AB, FF and MR partecipated the molecular and biochemical studies. AC, DT, SO and MC carried out the clinical evaluation of the patient and wrote the patient case. SC and PC carried out the neurological studies.

MS and MM carried out the histological analysis. GPC, AB, PV conceived of the study, partecipated in its design and coordination and helped to draft the manuscript. All authors read and approved the final manuscript.

## Pre-publication history

The pre-publication history for this paper can be accessed here:

http://www.biomedcentral.com/1471-2377/11/85/prepub
